# Independently testing prosocial interventions: Methods and recommendations from 31 researchers

**DOI:** 10.1111/nyas.15393

**Published:** 2025-06-27

**Authors:** David J. Grüning, Julia Kamin, Emily Saltz, Tin Acosta, Dominic DiFranzo, Beth Goldberg, Alex Leavitt, Filippo Menczer, Tyler Musgrave, Yixue Wang, Magdalena Wojcieszak

**Affiliations:** ^1^ Psychology Department Heidelberg University Heidelberg Germany; ^2^ Department for Survey Methods and Design GESIS ‐ Leibniz Institute for the Social Sciences Mannheim Germany; ^3^ Prosocial Design Network New York City New York USA; ^4^ Civic Health Project Palo Alto California USA; ^5^ Jigsaw, Google LLC New York City New York USA; ^6^ Department of Computer Science and Engineering Lehigh University Bethlehem Pennsylvania USA; ^7^ Roblox San Mateo California USA; ^8^ Observatory on Social Media Indiana University Bloomington Indiana USA; ^9^ School of Information University of Michigan Ann Arbor Michigan USA; ^10^ Technology and Social Behavior Program Northwestern University Evanston Illinois USA; ^11^ Department of Communication University of California Davis California USA; ^12^ Center for Excellence in Social Science University of Warsaw Warsaw Poland

**Keywords:** digital intervention, expert convening, future actions, methods, prosociality, recommendations

## Abstract

There is a growing need for independent, ecologically valid research on prosocial design interventions within online platforms. Platform design has a significant impact on user interactions, yet independent researchers often lack access to live platforms, limiting the ecological validity of studies testing the effectiveness of prosocial interventions. In response, 31 experts from academia, industry, and civil society gathered for a workshop focused on understanding methods that enable effective research on design interventions that lead to prosocial outcomes, such as healthy interactions and individual safety, well‐being, and dignity. We synthesize the workshop findings to guide researchers and practitioners in advancing prosocial design and to provide stakeholders—platforms, advocacy groups, and regulators—with evidence‐based tools to promote healthy online behavior. First, we present a review of the dominant research methods in the field. Second, we highlight the challenges that researchers agreed were most prominent (such as reinvention of the wheel) for testing interventions and recommend ways to address them. Finally, we propose specific future actions to address the challenges in the areas of knowledge sharing, events, and supporting infrastructure that experts consider most worth pursuing, and set out an agenda for future online prosocial research and investment.

## Graphical abstract

Independent research plays a critical role in producing knowledge around prosocial design, generating evidence that platform designs can create positive outcomes, and pointing to the most effective design patterns. Such knowledge can suggest specific design solutions to platforms, while also providing a lever to advocacy groups and regulators to externally push for reforms. This paper builds on and substantially expands other calls to support independent research to accumulate the collective knowledge around prosocial design.

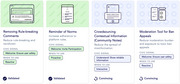

## INTRODUCTION

Online platforms are designed spaces. Like any space, their design choices shape users’ experiences. A growing number of independent researchers in civil society, academia, and industry groups are studying, developing, and testing prosocial designs. For the purposes of this convening, we define prosocial interventions as the set of design patterns, features, and processes, that foster healthy interactions between individuals and create the conditions for those interactions to thrive by ensuring individuals’ safety, well‐being, and dignity. Prosocial interventions may include testing or promoting platform features that actively promote prosocial behavior (e.g., guidance for posting^1^), those that make it easier for users to act prosocially by removing barriers that get in the way (e.g., guidance on how to handle harassment or confront it^2^), and also those features that prevent or thwart harmful or antisocial behavior (e.g., removing rule‐breaking comments ^3^).

How can we tell what is working and what is not? A core challenge facing any independent researcher when aiming to assess the effects of their designs on users’ exposure, behaviors, and/or attitudes is that they do not usually have access to platforms on which these effects can be tested. As a result, many independent studies are not conducted in environments that are representative of a real user's context and information ecosystem; in research parlance, they lack ecological validity. This means that even as hundreds of prosocial intervention studies are published every year, testing everything from fact‐checks to bot assistants, researchers often have little insight into their generalizability and potential real‐world impact.

There are, however, a number of approaches researchers use to move toward greater ecological validity, such as observational studies using public platform data, browser extensions that modify users’ online experience, mobile applications, or simulated social media platforms with varying levels of verisimilitude. These approaches present opportunities for independent research but also come with their own set of limitations and challenges, which can include the development and maintenance of production‐ready software, access to platform data, and the costs of recruiting participants. Given those challenges, there exist considerable barriers for their use, especially for junior scholars or scholars from less well‐resourced institutions. Moreover, given the fast pace of change of tech platforms and of the state of the art of conducting independent research, it is difficult for researchers to assess the relative advantages and constraints of their methodological options.

To address these challenges, on December 1st, 2023, 31 researchers from across academia, industry, and civil society were convened by Prosocial Design Network (PDN) and Jigsaw for a day‐long workshop to share knowledge, map the landscape of ecologically valid approaches to test prosocial interventions, and explore opportunities for researchers to collectively accelerate independent, ecologically valid research around prosocial design (see Table S1 for the convening plan in detail). Most participants were from US‐based institutions (see Table S2).

In this paper, we share the two main outputs of the convening: (1) a synthesis of the main approaches in the research space discussed at the convening, including the strengths and limitations of each, along with brief summaries of additional approaches; and (2) the central recommendations from the event. We also share future actions that other researchers and practitioners can take to impact the field in the most meaningful way.

Independent, ecologically valid research of prosocial design is increasingly critical. Social media platforms have the capacity to create harm for individuals and society^4^, ^5^, ^6^, ^7^, ^8^, ^9^ but can also integrate design that both reduces that harm and creates spaces for human flourishing. Platforms face constraints and do not always have the incentive to research and share knowledge around effective prosocial design. While industry–academia research collaborations can fill that gap in part (e.g., Refs. 10 and 11), opportunities for those collaborations are rare and often face obstacles.^12^ Independent researchers play a critical role in producing knowledge around prosocial design, generating evidence which platform designs can create positive outcomes, and pointing to the most effective design patterns. Such knowledge can provide platform‐specific design solutions, while also providing a lever to advocacy groups and regulators to externally push for reform. This paper not only builds on but substantially expands other calls to support independent researchers to build our collective knowledge around prosocial design.^12^, ^13^, ^14^, ^15^, ^16^

## WHAT ARE INTERVENTIONS FOR PROSOCIAL DESIGN?

**FIGURE 1:**
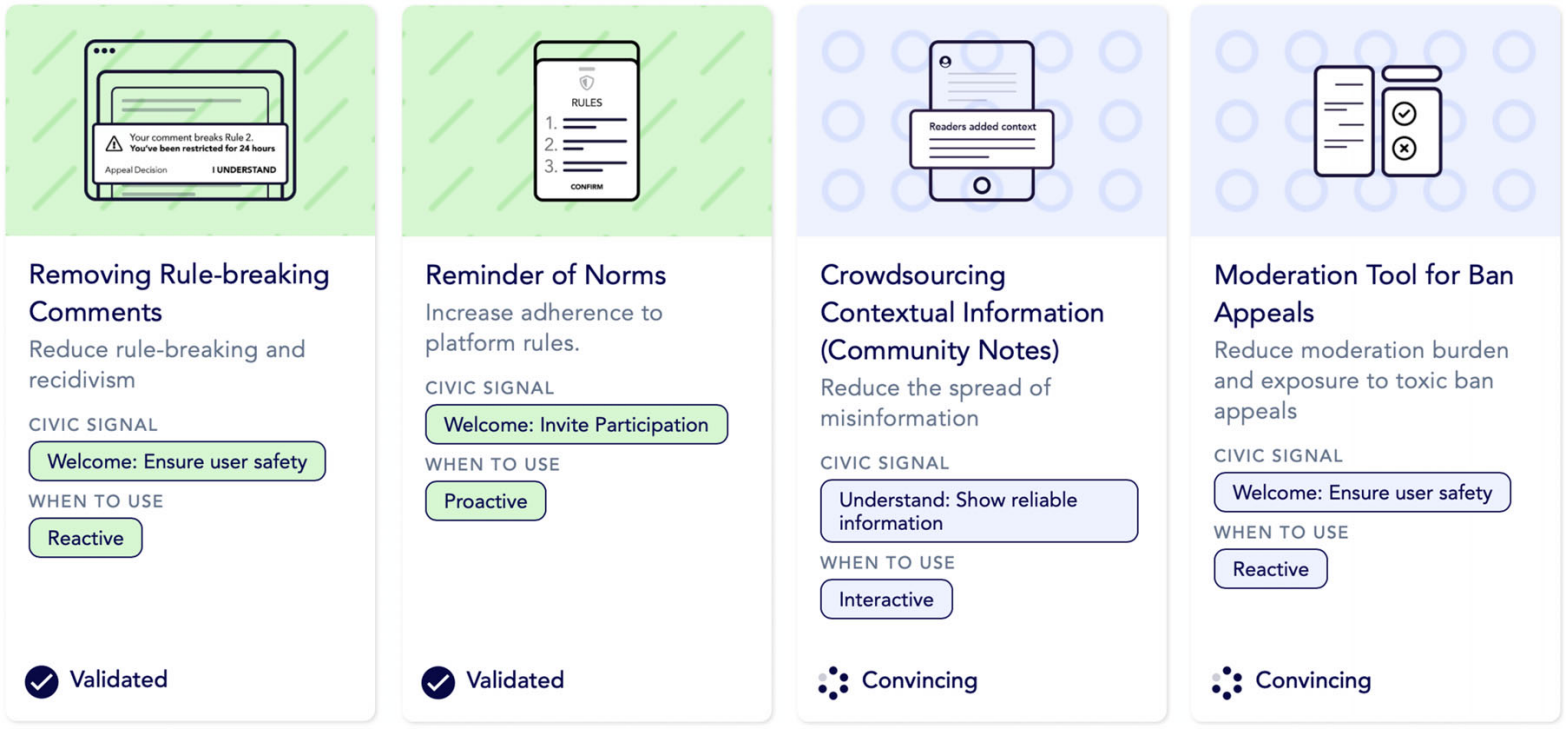
Exemplary intervention entries from Prosocial Design Network's evidence‐based library.

The range of interventions referred to in this paper are ones that are digital, scalable, automated, and tested,^17^ and with the expressed purpose of either addressing online harms or promoting positive social and societal outcomes directly. The Prosocial Design Network evidence‐based Intervention Library (www.prosocialdesign.org) lists dozens of examples of previously explored intervention types meeting these criteria with various degrees of evidence, and addressing a variety of prosocial outcomes, including reminder of norms, accuracy prompts, highlighting consensus posts, and labeling misleading content. Figure 1 shows a section of the library entries. It is important to note that, to date, the field of digital prosocial intervention research has mostly focused on reducing harm and removing barriers for users to act prosocially online. Notably, however, the focus is increasingly shifting toward research programs that develop and test interventions that directly encourage digital prosocial behavior, either by guiding users toward prosocial behavior (e.g., guidance on posting^1^) or by providing them with a toolset to act prosocially (e.g., an upvote button^18^).

## METHODS OF DIGITAL INTERVENTION RESEARCH

**FIGURE 2:**
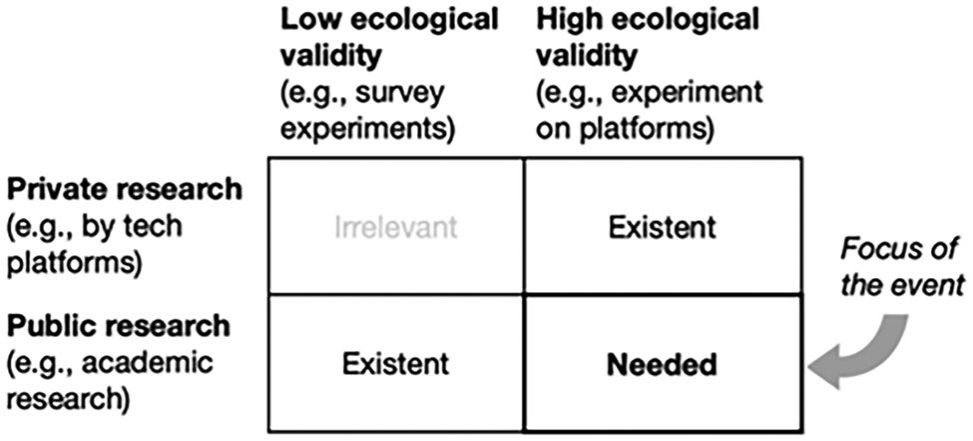
Overview of methods commonly employed by public and private researchers.

There are many independent studies that test the effectiveness of digital interventions, but only a fraction of them show high ecological validity, for example, testing in the field on live platforms. In an analysis of 20 years of trust and safety research, Dratver and Katsaros^19^ compiled an estimate of ecologically valid studies and found that a small minority were experimental, with only 2% conducted in the field. In addition, platforms test interventions regularly and intensively but mainly do so behind closed doors, only sometimes reporting curated results (e.g., Refs. 20, 21). The gap in intervention research methods that are both ecologically valid and conducted in public by independent institutions is highlighted in Figure 2.

The workshop aimed to address this gap. It began by discussing what the organizers identified as promising methods for independent scholars to do more ecologically valid research: browser extensions and mobile apps, simulated platforms, and observational studies. We outline these methods in detail below, followed by an overview of other strategies for intervention testing that emerged in the workshop, as summarized in Table 1.

**TABLE 1:** Summary of methods discussed in this paper.

Method	Description	Prerequisites	Strengths	Limitations
Browser extensions and mobile apps	Software that alters users’ online experience on platforms (e.g., Facebook, X/Twitter) or mobile devices.	• Engineering resources to implement extensions or apps	• High ecological validity • Direct observation of behavior and attitudes • Flexibility in intervention design	• Costs (software, recruitment) • Recruitment limitations (convenience samples, self‐selection bias) • Software maintenance • Users currently can not access extensions on native mobile apps • Potential legal challenges from platforms
Simulated platforms	Simulated online environments that mimic existing or new platforms, and test the effects of design choices.	• Design and engineering resources to create a usable platform	• Controlled environment • Isolation of variables • Replicability • Reduced potential harm to users	• Variable ecological validity • Lack of real‐world social networks • Resource intensive • Difficulty in recruiting representative samples
Observational studies (natural experiments and correlational studies)	Utilize existing platform data to study the effects of interventions already implemented on platforms.	• Access to platform data (APIs, datasets, scraping)—Identification of proxies for random assignment (for natural experiments) • Large sample of subgroups with variation in feature use (for correlational studies)	• High ecological validity • Focus on real‐world interventions • Relevance to platform practices	• Lack of true random assignment • Data access limitations and restrictions • Potential legal risks with web scraping
Other methods	• Collaborative field experiments with online communities • Recruiting participants and nudging their on‐platform behavior • Sock puppet/user bot studies • Using ads infrastructure for intervention delivery and measurement • Simulating social media with LLMs as participants	Vary depending on the specific method.	Vary depending on the specific method.	Vary depending on the specific method.

## BROWSER EXTENSIONS AND MOBILE APPS

### Description

Browser extension and mobile app studies both make use of software that is downloaded by subjects onto their desktop or mobile device and then can be used to alter subjects’ online experiences. Researchers can deploy this software to alter design features on platforms to observe how different interventions affect subjects’ behaviors and attitudes. This can be done either by monitoring behaviors online longitudinally or by conducting surveys on or off the platform, all with the research subjects’ informed consent.

**FIGURE 3:**
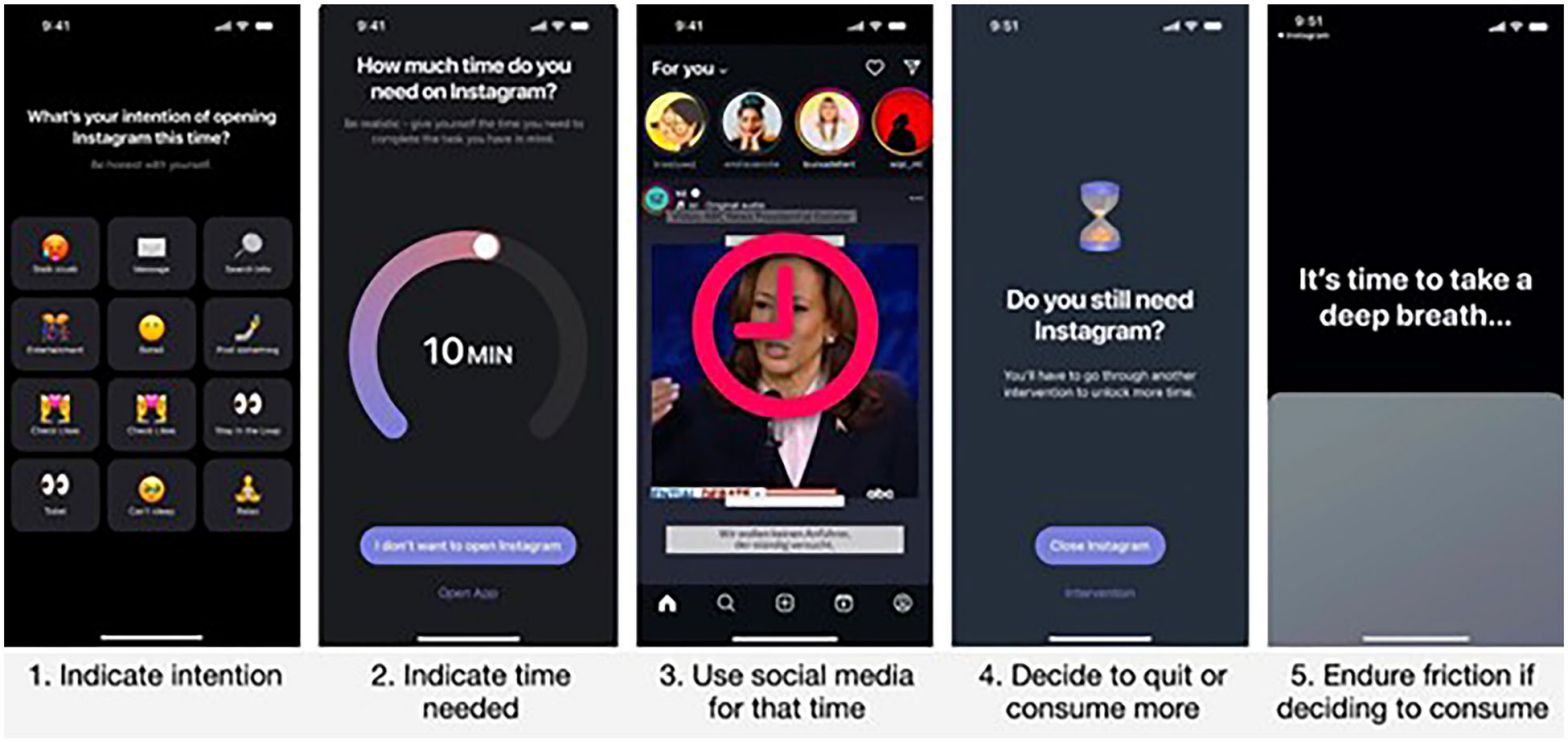
Intervention set‐up by smartphone application one sec.

Currently, browser extension studies are distinct from mobile apps in that they can alter the design and experience of social media platforms (e.g., Facebook, X/Twitter). For example, Beknazar‐Yuzbashev et al.^22^ used a browser extension to remove posts with toxic content from recruited subjects’ Facebook and Twitter feeds, observing the impact on their behavior and well‐being compared to a control group. Similarly, Yu et al.^23^ developed a browser extension to increase recommendations and exposure to verified and ideologically balanced news in users’ YouTube ecosystem. Existing mobile app studies do not alter subjects’ experiences *within* social media apps; they instead create external nudges intended to alter subjects’ use of other apps on their mobile devices. For example, experiments conducted on the research app *one sec* test approaches—like reminders, friction, and options to dismiss—to reduce subjects’ unwanted use of distracting apps^24^, ^25^ (see also Refs. 26, 27), as shown in Figure 3. Studies with the two technologies also vary on how much they depend on voluntary adoption of the tools, by providing a useful tool versus needing to actively recruit and pay subjects.

### Prerequisites

These studies tend to be technologically demanding and costly. In the case of browser extension studies, one challenge is building software that not only seamlessly alters a user's platform experience but does so without otherwise diminishing that experience by, for example, adding load time. This method also requires user experience expertise to design and implement new interventions in a way that is usable enough to be adopted by extension or mobile app users. Studies frequently recruit users via online ads, which, in particular for extensions and apps that do not provide a useful product, can be a greater expense than software development. Those that recruit participants from proprietary online panels (e.g., YouGov, Prolific) also face costs that are often prohibitive to smaller or less well‐resourced labs or institutions.

### Strengths

Browser extension and mobile app studies present possibly the best opportunity for independent, ecologically valid research in terms of testing users in a naturalistic setting. By conducting studies on the platforms that subjects use, these studies can directly observe how a design or algorithmic change on social media can change a subject's online behavior or, via surveys, their beliefs and attitudes. Mobile app studies similarly can directly observe how an intervention on subjects’ mobile devices can shift their behavioral patterns.

### Limitations

In addition to costs, recruitment presents a limitation to these studies by largely depending on convenience samples or those from private market research companies. Recruitment via social media ads has the benefit of targeting frequent platform users but also introduces a self‐selection bias toward users willing to download a browser extension, as well as potential bias from a platform's optimization algorithms. In turn, samples recruited from proprietary online panels may not represent heavy social media users. In addition, researchers express a concern that there may also be a natural limit to the number of users willing to engage in these types of studies. Mobile apps, particularly those that offer a useful service, may expand the potential subject pool, but those subjects will be an even more selective set, that is, users who are open or even eager to change their online behavior.

Additional limitations include maintaining software to be current with platform changes that can break how an extension interacts with website content and integrating data practices and systems to secure the privacy of participants. As discussed above, because mobile apps currently are unable to alter the design of social media apps, studies that depend on direct changes to platform design will exclude mobile‐only users. Finally, browser extension and mobile app studies may face legal challenges from platforms.^16^ As of this writing, at least one case has been adjudicated to determine to what extent platforms limit the use of third‐party extensions to alter users’ experiences.^28^

### Takeaways and opportunities

For conducting ecologically valid field experiments, browser extension and mobile app studies offer perhaps the best alternative to collaborating on research with platforms. At the same time, they come with perhaps the greatest costs, both in terms of software development and recruitment. Recruitment also poses limitations both in terms of subject representativeness and, possibly, a natural limit to the number of subjects willing to participate.

Software and recruitment costs have the potential to decrease, however, as social scientists pool knowledge and software development, sharing best practices for recruitment and making software code available to other researchers. Open‐source repositories—ideally that are maintained, have systems to prevent use as malware, and provide guidance—can reduce the time and money researchers need to invest in software development. As of this writing, several projects have made such resources available, for example, the browser extension Webmunk (www.webmunk.org) and the interventive smartphone application *one sec* (https://one‐sec.app/). Similarly, researchers can save time and expense by building a best‐practices repository on the most effective ways to recruit participants.

Deeper levels of collaboration and coordination could result in even greater collective gains. For example, a research center could maintain a service that would provide the base browser extension functionality that researchers could modularize to specific studies. That research center could also facilitate recruitment or even maintain an ongoing panel of subjects who use the browser extension and then opt into studies.

## SIMULATED PLATFORMS AND LLM TEST BEDS

### Description

Simulated social media platforms bypass the need for direct platform testing by simulating existing or new types of online platforms. These provide researchers with controlled environments for researching the effects of online interventions. They can be implemented in various ways, differing in dimensions like platform resemblance (e.g., near‐exact clone of an existing platform, a generic platform, or new model), interaction capabilities (e.g., clickable prototypes without dynamic input, or live input with dynamic engagement), and user bases (from prescripted mock users to simple rule‐based agents, to personas and messages generated by large language models (LLMs)). In an alternative approach, both the environment and study subjects are fully simulated, to study a model of an environment without any human users involved.^29^ We outline the simulation approach as an additional method in the section “Other notable methods for independent intervention research” below.

**FIGURE 4:**
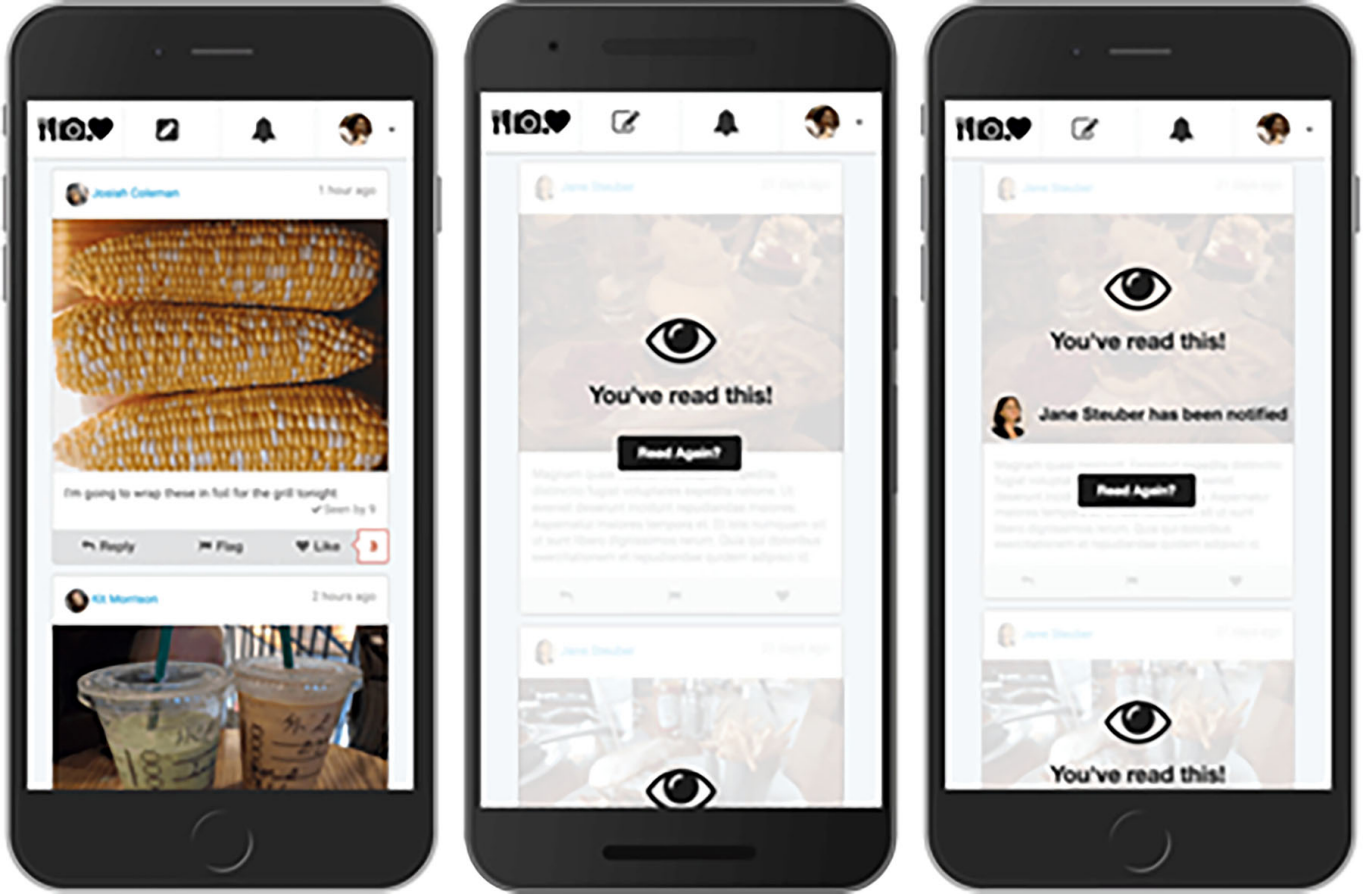
The Truman platform, an open‐source social media simulation.

Examples of simulated platforms include the open‐source Truman platform from Cornell's Social Media Lab (github.com/cornellsml/truman_2023), shown in Figure 4, which can be used to create bespoke platform environments for studies, such as Social Media TestDrive;^30^ the Social Media Accelerator, a collaborative environment for designing experimental research, modeled on CERN,^31^, ^32^ an open‐source platform and architecture for social media experiments; and Deliberate Lab (github.com/PAIR‐code), a platform for running online research experiments on human and LLM group dynamics, open‐sourced by Google's People+AI Research (PAIR) Initiative.

### Prerequisites

Simulated environments can range in fidelity and interactivity, with different levels of skill and effort needed by the researchers. On the low end of fidelity, platform context can be represented in static wireframes, sketches, edited screenshots, or mocks, which may require some minimal knowledge of design software. For more interactivity, mocks can be weaved together in a clickable prototype to resemble platform behaviors. This approach is most accessible, and thus widely employed by UX and HCI practitioners for its speed and low cost. For more complex mobile apps^24^ or web environments like Truman,^30^ in‐depth programming knowledge is required. These often require researchers to allot significant time, effort, and budget to customize an environment for a given study.

### Strengths

Simulated platforms and content allow researchers to create realistic social media experiences while isolating variables for precise analysis. These platforms enable full control over experimental treatments, facilitate replication of studies, and offer the flexibility to experiment with platform features and designs. Importantly, they also reduce potential harm to users compared to real platforms, making them safer for conducting sensitive research.

### Limitations

The degree of ecological validity across simulated platforms can vary widely, as the balance between realism and complexity often leads to trade‐offs. Even if simulated platforms are complex in their design and aim to replicate existing platforms, study participants are fully aware that they are not on a real social media platform and may use the mocks very differently than they would an actual platform. Participants also lack their actual social network, which cannot be simulated, and which leads to very different user interactions and experiences, minimizing ecological validity. Thus, the overall artificial nature of the platform and the lack of a genuine social network raise the questions of how much more valid simulations really are compared to alternative methods, even if the environment is superficially similar to a live platform at a point in time.

Next, while these simulations offer researchers immense control, they also suffer from decentralization and lack of common standards (an issue outlined in the section on meta‐learnings below). Since each one is developed independently, this leads to both high costs for similar infrastructure and difficulty in making comparisons and assessments across different simulated environments. Adding to the design and engineering resource needs mentioned previously, recruiting representative samples is also difficult, and maintaining plausibility for longitudinal studies can be problematic if the platform is posing as a real one. Even when people are on a simulated platform, there is the issue of incentivizing users to actually engage in the use of a platform without authentic social rewards, as noted by platforms like Truman.^30^

### Takeaways and opportunities

In summary, simulated platforms offer a controlled environment for studying online behavior, allowing researchers to isolate variables and design their own experimental tests of platform features. They provide advantages in terms of researcher control and replicability. However, limitations include their ultimate artificiality and lack of real‐world social networks, and the resource‐intensive nature of developing, maintaining, and analyzing data across such platforms. Some of these limitations noted could be addressed by more coordinated efforts by researchers and platforms to make simulations easier to create and analyze.

Opportunities include creating quick‐start environments, interoperable standards and formats, and creating generative content and algorithms aligned with live platform data.

While some efforts are already underway to create quick‐start environments, such as YourFeed,^32^ the research ecosystem would benefit from more robust open‐source, ready‐to‐use options for simulated environments and content. Perhaps just as important is the need for interoperable standards and formats for such platforms. Greater awareness and cooperation between researchers creating mock platforms could enhance research outcomes, and establishing a consortium or network of simulated platforms could standardize infrastructure and data formats. Standards for interpreting and reporting findings, as well as an archive for study results, could ensure consistency and accessibility. Finally, fostering interoperability through platform APIs and sandboxes and updating user content, and platform‐specific features such as feed ranking algorithms based on real platform data to inform generated user and content, could bridge the gap between simulation and real‐world applications, operating similar to staging environments used within platforms.

## OBSERVATIONAL STUDIES: NATURAL EXPERIMENTS AND CORRELATIONAL STUDIES

### Description

Observational studies include natural experiments (or quasi‐experiments) and correlational studies, both of which make use of existing platform data to draw causal inferences about the effectiveness of interventions currently used on platforms.

Natural experiments take advantage of phenomena that can serve as a substitute for random assignment of treatment (i.e., the use of a design pattern) to model a post‐hoc quasi‐experiment. Random assignment proxies can take different forms. Renault et al.^33^, for example, make use of a threshold which determines when a community note is deemed useful, and so becomes visible to users, to infer how much adding a public Community Note decreases the retweet rates of misleading content. Another common approach is to use difference in differences models to measure the impact of initiating or discontinuing a prosocial feature, a strategy used by Papakyriakopoulos et al.^18^ in observing the effects on discourse quality when subreddits discontinued Up and Down votes. Yet other methods include matching treated observations with untreated controls^34^ or taking advantage of delays in treatment^3^ (see also Refs. 35, 36).

Observational correlational studies do not use proxies for random assignment but instead leverage variation in the use of a feature across subgroups on a platform, which allows for comparison of the effect of a feature. Studies using the approach will often, if not always, control for other relevant variables of those subgroups. Jhaver et al.^37^, for example, analyzed data from millions of posts across the Reddit platform, to observe the effects of if and how users are offered explanations when their comments are removed, which varies across the platform's thousands of subreddits.

### Prerequisites

First and foremost, the researcher will need access to platform data. In many cases, those data will be collected via APIs that platforms provide or, in special cases, when platforms make large datasets available to researchers. If no API or publicly shared datasets exist, researchers may scrape data using automated methods that draw data from a platform's site, for example, using HTML tags, from the platform's site. In some cases, researchers will collect vast stores of data which they in turn share with other researchers.

To construct a natural experiment, the researcher will also need to identify a phenomenon that can act as a substitute for random assignment. As discussed above, such proxies for random assignment can take many forms, including but not limited to the rollout—or rollback—of a feature, thresholds that determine when an intervention is executed, matched controls, or delays in treatment.

In the case of correlational studies, the researcher will need observations across a large sample of subgroups where there is variation in the use of design features, for example, subreddits and Facebook groups.

### Strengths

The strength of this method lies in its ecological validity; researchers can observe the effects of treatment in the wild, with real users in a setting that includes all the complex interactions of a real platform, by virtue of being a real platform. A related inherent strength of this method is that its focus will necessarily be on a feature that platforms use, guaranteeing the relevance of the research.

### Limitations

In addition to the constraints discussed above (i.e., researchers are limited by access to data and either the existence of a proxy for randomization or of variation across groups or communities within platforms), the main limitation of this approach is the absence of true random assignment. For both natural experiments and observational studies, there will be a certain level of endogeneity in comparing treatment to control. Even in well‐chosen proxies with random assignment in natural experiments, there may be hidden confounding variables that account for when an intervention is executed. Even more so in the case of correlational studies, it should be assumed that the use of a feature across subplatforms is not random. In both cases, using matching techniques (i.e., pairing individual observations that match each other almost identically save for the treatment) and controlling for potential confounding variables in regression analyses, as researchers most often do, should mitigate those endogeneity concerns but not entirely.

Researchers must also contend with unknowns, risks, and an ever‐shifting landscape associated with data availability.^16^, ^38^ APIs and data repositories that platforms build for researchers are in a constant state of flux, with the trend being toward less access and more restrictions, although regulatory pressure, for example, from the EU's Digital Services Act, could reverse that trend^39^. Researchers must also rely on trust that the platforms’ data sampling is unbiased, and that no other confounding variables are hidden from access. As of this writing, researchers who resort to collecting data independently via web scrapers also face legal risks posed by companies which challenge that data collection violates their terms of service (e.g., see Ref. 16 for a review of the legal and ethical challenges of web scraping).

### Takeaways and opportunities

Observational studies offer perhaps the approach with the strongest ecological validity to independently study the effects of prosocial interventions; by observing the impact of interventions on platforms, researchers study an intervention that platforms have rolled out and can account for all the complex variables and interactions included in implementing the intervention in the real world. Their strength in ecological validity is counterweighted by the relative weakness in the internal validity of observational studies; absent true random assignment, researchers cannot completely discount possible confounding variables that might explain the observed effects. A greater limitation to this approach, however, may be access to data and, in particular, access to data that either gives researchers the ability to make use of a proxy for random assignment or that allows them to observe variation in the implementation of a design intervention. Future regulatory changes, however, could reduce that limitation.

## OTHER NOTABLE METHODS FOR INDEPENDENT INTERVENTION RESEARCH

While the three approaches to independently test the effectiveness of prosocial intervention discussed above are perhaps the most promising with regard to ecological validity and scalability, they are not an exhaustive list. Researchers use several other approaches to experimentally test potential interventions in ecologically valid settings. These other approaches include conducting field experiments in collaboration with online communities; deploying interventions as ads on platforms; nudging the behavior of users indirectly; and using sock puppets to deliver interventions. Each is described below.

### Collaborative field experiments with online communities

Members of online communities, including moderators and volunteer hosts of online group forums, can be partners in designing and conducting field experiments to test online interventions. That is the case when, for example, subreddit moderators have both the capacity to implement and test new interventions as well as generate enough engagement among users to be able to conduct randomized trials with adequate sample sizes. Examples of collaborative field experiments with communities include studies on the impact of posting sticky notes reminding users of community norms in subReddits^40^, and the effect of thanking editors on Wikipedia.^2^ Collaborative studies often include a process of codesigning interventions with communities.^41^ While such studies have the dual benefit of having high ecological validity and providing immediately relevant knowledge to the custodians of the internet, they also present challenges for the researcher and community; it may take considerable time for researchers to cultivate the trust required to enter a collaboration, and studies will require both considerable time from community members as well as additional care in designing experiments that minimize potential harm to the community.

### Recruiting participants and nudging their on‐platform behavior

Yet another approach approximates the effect of a platform intervention by first recruiting participants and then randomly assigning nudges to change their on‐platform behavior. Levy^42^, for example, tested the potential impact of recommending diverse news sources to users by first recruiting Facebook users to participate in a survey and then, using a Facebook plugin integrated into the survey, nudging them to like and follow political news accounts. Bowles et al.^43^ took a similar approach, inviting recruited participants to receive Whatsapp messages prebunking false news narratives (see also Refs. 44 and 45). The advantage of this approach is that researchers can alter the experience of users without needing to work with platforms and with little technological development. There are several limitations, however, including that researchers are confined to treat only users willing to participate in a study. These studies also tend to rely on survey responses, as opposed to changes in online behavior, to detect effects that constrain statistical power. A related set of browser apps are used to test strategies to improve how users engage on social media platforms, for example, the Fakey app and the Bad News Game, which both use gamification to build skills in identifying misinformation.^46^, ^47^

### Sock puppet/user bot studies

Yet another way to treat platform users and alter their online experience is to create fake accounts that interact with users—by, for example, following them, commenting on posts, or messaging them—to deliver a treatment. Munger^48^, for example, created user accounts on Twitter that nudged other users via comments to use less offensive language. Askari et al.^49^ created LLM‐trained bots that replied to Twitter users to encourage them to engage with verified and ideologically balanced news sources. In these cases, researchers randomly assigned which of those real users received treatment and then observed the subsequent online behavior of those users. The strength of this approach is that researchers can observe real effects on real platform users. The main limitation is imagining and knowing how to translate these interventions into scalable platform designs. Specifically, any self‐disclosed bot used by platforms would lack the peer influence effects of real users and, depending on the platform, may even have a negative valence. Furthermore, using bots that pose as users likewise presents ethical issues for both researchers (although attenuated by an institutional review board) and platforms. Finally, similarly to data access restrictions, platforms make it ever more challenging for researchers to create sock puppets or user bots, making such experiments increasingly challenging. An alternative may be to encourage real users to intervene, for example, by proactively shaping the discourse of users (e.g., through digital badges^50^).

### Using ads infrastructure for intervention delivery and measurement

Finally, researchers can make use of ads and other third‐party affordances on platforms to treat users with nudges, educational boosts, and other messaging interventions. Lin et al.^51^, for example, treated X/Twitter users by targeting them with ads reminding them of the importance of accuracy, and then observing users’ propensity to share false news stories compared to a control group. Other third‐party features like polls could be similarly leveraged. Using ads and other platform third‐party features gives researchers a promising way to test interventions without some of the legal and ethical concerns or other approaches. However, they introduce challenges both at the point of assignment and in data collection. Because platforms generally use obscure optimization algorithms to decide who is shown an ad, researchers may not be able to assume treatment is truly randomly assigned. Collecting data to observe behavioral treatment effects either requires public data access, as is the case for observational studies, or that researchers recruit participants and gain their permission to collect their data, either via surveys, apps, or browser extensions. Researchers have found ways to work around these challenges, for example, assigning treatment by geographical district^52^, ^53^ or by using in‐platform polls to collect survey data.^54^

### Simulating social media with LLMs as participants

A variation of the simulated social media approach (discussed above) is to create fully simulated environments in which the subjects in the experiment are LLM agents.^55^ This approach leverages the ability of LLMs to absorb context and make decisions with growing similitude to humans.^56^ Its advantage is that it removes one of the largest constraints on conducting experiments, that is, recruiting participants. The use of LLMs to simulate user personas and their behaviors, however, faces limitations. For one, models only estimate true human behavior and may undermine the validity of an experiment due to bias in the underlying training data. Similarly, it is not clear whether LLMs can be used to build representative samples of a population based on demographic descriptors.^57^, ^58^, ^59^ However, fully simulated social media environments could contribute to research in this space as an intermediary step, allowing researchers to conduct experiments at scale to pre‐test and identify promising designs that might then be tested with human participants.

Although not a focus of this article, it is also important to call attention to the critical role that qualitative research plays in understanding which interventions will work in real‐world contexts. Qualitative methods, including ethnographic studies and user surveys, play a central role in identifying, generating, and understanding potentially effective interventions. In the second part of this article, we briefly discuss how qualitative and quantitative research are both essential in a multimethod approach to studying prosocial design.

## IMPORTANT RECOMMENDATIONS FOLLOWING FROM THE EXPERT EVENT

The workshop highlighted shared challenges and learning across studies and disciplines. We outline the key observations that provide an understanding of the challenges and recommendations for other researchers and practitioners of prosocial design.

### Building centralized and shared resources

The most frequently discussed and strongly agreed upon issue in the field of digital intervention research was the problem of resource fragmentation. Specifically, digital intervention research is by default linked to a variety of methodologies and technologies, ranging from randomized controlled experiments to experience sampling and behavior tracking via mobile applications. Although methodologies are described to some extent alongside existing published research, detailed guidance on resources and development for these methods or tools is largely missing. As a result, researchers are constantly reinventing the wheel, building methodological frameworks and technical tools that have already been built in the same or at least similar ways.

In addition, many methods are hindered in their practical implementation by a researcher's limited technical and financial resources. This is especially true for computationally intensive intervention designs such as simulated platforms and browser extensions, as well as entire smartphone applications. Many experts pointed out that there is no resource (e.g., a website or repository) that shares tools and insights for researchers to build their own designs and test interventions. Notably, this lack of resources favors better‐resourced labs and institutions, where researchers have the funds to hire software developers or recruit large quota samples, creating inequality.

To address this state of methodological fragmentation and differing institutional resources, we propose collaborations between researchers and practitioners to develop specific tools and agree on standards for more ecologically valid intervention testing. These collaborations enable people to share experiences and expertise regarding different methods and tools firsthand. Collaborations may also aim to produce resources for others. These resources could include (1) information on existing methods relevant to the specific intervention on which a researcher wishes to focus, (2) the strengths and caveats of using these different methods, and even (3) concrete parts (technical and conceptual) to build the intervention method as well as a knowledge base for how to adapt the method. More ambitiously, such resources can be made openly accessible to any interested researcher. Such open resourcing can be reinforced by communities creating platforms for external researchers to add and contribute new methodologies and tools (e.g., digital libraries for intervention approaches and GitHub repositories for specific experimental programs to run).

### Promoting common standards for methods

One of the critical issues in the field of digital intervention research, particularly in relation to methodologies such as browser extensions, intervention applications, and simulated platforms is the lack of standardized approaches. Despite the increasing prevalence of these digital interventions in both experimental and applied research, there is still a lack of consistent, universally accepted protocols to guide the development, implementation, and evaluation of these tools. This deficit not only complicates the replication of studies but also hinders the ability to compare results across studies and disciplines. Furthermore, this lack of standardization extends to the evaluation of digital interventions. The metrics used to assess the effectiveness of tested interventions vary widely between studies, with little consensus on best practice. This variability limits the generalizability of findings, making it difficult to draw broad conclusions about the effectiveness of digital interventions in different contexts.

Establishing common standards would not only streamline the research process but also improve the reproducibility and scalability of digital interventions. Such standards would allow researchers to build on one another's work more effectively, leading to faster advances in the field and more meaningful comparisons across studies. By promoting a more structured and collaborative approach to digital intervention research, the field can better address the complex challenges associated with implementing and evaluating these innovative tools.

### Understanding digital spaces as individual spaces

Another future goal for intervention research should be to dissect the complex interactions between digital spaces and their individual users, as these can moderate how effective a digital intervention is.^a^ That is, while a platform may provide roughly the same affordances and features to all its users, those users are bound to use these features in different ways, with different intentions, and—consequently—with different effects. For instance, although two users may use the same features on the same social media platform, they may receive substantially different content to consume, based on their past on‐platform behaviors and content consumption. Some researchers have provided tools to address these inter‐individual effects of digital platforms in the form of frameworks (e.g., Refs. 60, 61) or by gathering initial empirical evidence on individual differences in responses to prosocial digital interventions (e.g., Ref. 62). However, much research is still needed by the community, first to understand the intricacies of individual social media use, and second to design interventions that effectively adapt to a user's circumstances.

### Developing multimethod approaches

One insight that resulted from the discussion of different methods for independent research on digital interventions was that there exists no gold standard method, inasmuch as all existing methods have unique strengths and weaknesses. In this regard, multimethod approaches show promise in digital intervention research by combining different methods, each with unique strengths and weaknesses, for a comprehensive understanding. No single method is flawless; instead, combining methods provides a well‐rounded perspective on complex issues. Quantitative approaches, though common, often miss out on nuances beyond their theoretical frameworks, potentially overlooking key changes in dynamic environments (e.g., Refs. 63, 64). Qualitative methods address this gap by exploring specific cases, uncovering hidden issues, and providing insights into user behaviors and attitudes, especially valuable in intervention design (e.g., Refs. 62, 65). However, qualitative findings may lack generalizability, a gap quantitative designs can be addressed by standardizing and validating findings across broader contexts. Employing a variety of methods not only generates fresh insights but also strengthens validation. For instance, while self‐reports may poorly capture behavioral frequency or degree, they add value by revealing users’ well‐being, mental health, and perceptions (e.g., effects of online behavior). Testing interventions through diverse methods allows for a robust assessment of impacts across varied aspects of digital interaction.

Practically, a phased approach can be used to combine methods and enhance the depth and applicability of a tested digital intervention. For example, to study an intervention aimed at promoting civil discourse, initial qualitative interviews with laypeople and experts can provide a conceptual foundation by defining civic discourse and identifying measurable indicators. Following this groundwork, controlled lab experiments can test the intervention's potential effects (e.g., increasing engagement in civic discourse). To account for real‐world complexity, researchers can then use simulated platforms for intermediate testing, allowing for adjustments based on factors observed. If successful, the intervention may finally be tested in a randomized controlled trial on a live platform, exposing it to the full complexity of natural digital interactions. Notably, at the core of a successful phasing approach sits the combined experience and expertise of researchers from different fields and their methodologies. Such matched expertise allows the team to follow through with the different phases, understand their different results, and correctly adapt subsequent phases based on the gained insights.

## OUTCOMES AND FUTURE WORK

### Landscape of worthwhile projects

**FIGURE 5:**
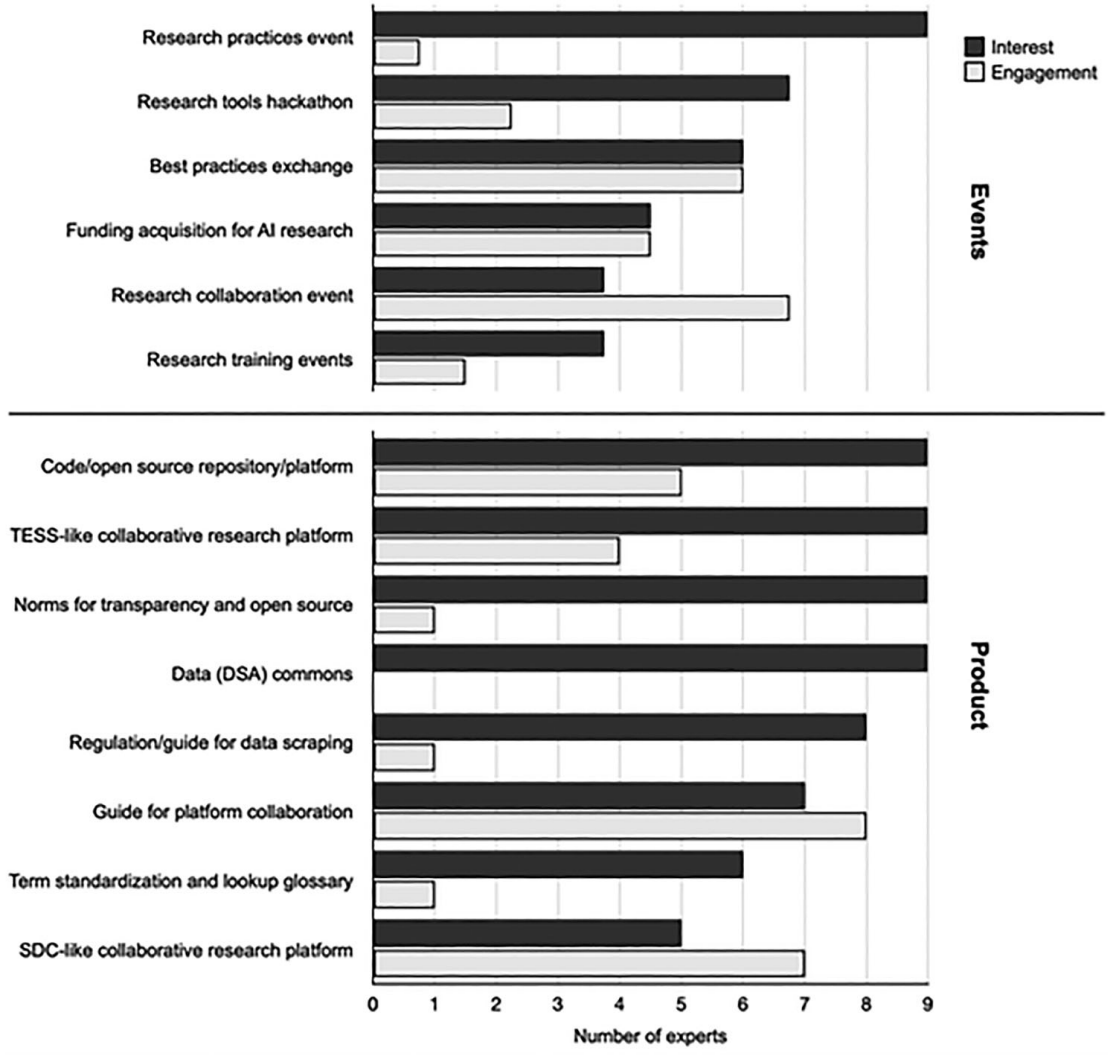
Survey on experts of digital intervention methodology indicating the most relevant next actions to be taken in digital intervention research.

Besides mapping and discussing prominent methods for digital intervention research, a main focus of the workshop was to identify projects needed to foster research in the digital intervention space. To do so, in the workshop, participants proposed and refined promising ideas for concrete projects. In a follow‐up survey after the event, participants then indicated their interest in (i.e., Which projects are you interested in being kept up‐to‐date?) and willingness to actively pursue these different directions (i.e., Which projects are you interested in helping to make happen?), resulting in a landscape of what experts in the field think are the most worthwhile projects to pursue. As shown in Figure 5, the most prominent projects (measured by the participants’ willingness to actively engage with them) concerned (1) the exchange of best research practices, (2) a guide for research collaborations with platforms, (3) organizing (recurring) events/a structure for building research collaborations, and (4) design‐focused research competitions (see, e.g., Strengthening Democracy Challenge, https://www.strengtheningdemocracychallenge.org). All actions are described in Table S3.

### Exemplary action underway

The PDN/Jigsaw convening already translated one of the most important agreed‐upon actions into a practical working group after the event, namely, the open‐sourcing of materials, designs, and tools for digital research. Specifically, a working group on browser extension research emerged from the event with a specific focus on open‐sourcing materials, designs, and tools to replicate and further develop existing digital interventions. The central aim is to align people's built extensions to avoid reinventing the wheel (e.g., open‐sourced software for conducting mobile digital intervention research). This group has already substantially grown beyond the original workshop participants, underscoring the interest of the community and the usefulness of this approach, and has lately produced an open resource on existing projects (https://github.com/louisbarclay/awesome‐web‐research‐tools).

Besides these immediate actions, all other specific actions are listed and explained in Table S3. They are categorized into (1) knowledge and resource sharing, (2) events, and (3) funding and infrastructure.

### Conclusions: Obstacles have to be overcome via community actions

To conclude, the workshop was an essential step in advancing the field of independent, ecologically valid research on prosocial interventions in online platforms. By bringing together diverse researchers and discussing methodologies like browser extensions, interventive apps, simulated platforms, LLM testbeds, and natural experiments and observational studies, participants developed a deeper understanding of the challenges and opportunities ahead. A prominent theme of the workshop was the challenges of translating each of the central methods into practically relevant research. We identified these major challenges, proposed ways to overcome them collectively, and also developed recommendations on how to move forward to advance not only the scientific knowledge on the issue of prosocial digital interventions but also the potential practical application of this knowledge to real‐world digital platforms and online spaces.

The collaborative efforts from this convening have set a foundation to address prominent obstacles of digital intervention research, prompting and organizing next steps in the form of new tools, open‐sourcing methods, and fostering partnerships that will further support prosocial design research and practical implementations on digital platforms.

## AUTHOR CONTRIBUTIONS

Review, discussion, and selection of content: All co‐authors. Conceptualization of manuscript content: D.J.G., J.K., and E.S. Writing the manuscript draft: D.J.G., J.K., and E.S. Reviewing and revising: All co‐authors.

## COMPETING INTERESTS

The authors declare no competing interests.

## PEER REVIEW

The peer review history for this article is available at https://publons.com/publon/10.1111/nyas.15393.

## Supporting information



Supplementary Materials

## Footnotes

a We note that this particular point was suggested in an insightful reviewer comment and did not stem from the original convening.
