# Supplementary material for: Independently testing prosocial interventions: Methods and recommendations from 31 researchers
Source: Ann N Y Acad Sci. 2025 Jun 27;1550(1):108–21. doi: 10.1111/nyas.15393 (PMC12412712; doi:10.1111/nyas.15393)
Supplement: Supplementary file 1 — Supplementary Materials [file NYAS-1550-108-s001.docx]

**Supplementary Materials**

**S1: Convening description**

To create a baseline of methodological understanding, the workshop agenda began with “deep dive” presentations from subject-matter experts on a subset of methodological approaches with promising ecological validity: 1) Browser extensions and interventive apps, 2) Simulated Social Media platforms and LLM testbeds, and 3) Observational studies, along with a presentation of additional approaches used in the field. Presentations were followed by breakout groups that reflected on the strengths and weaknesses of each method, which were then shared back to the broader group.

In the next part of the day, we broke out into groups to apply that knowledge on the strengths and challenges of each methods by designing research plans for interventions focused on four goals: (a) stemming misinformation, (b) reducing toxicity, (c) promoting meaningful dialogue, and (d) bridging divisions. Group participants used a template that included prompts to select the methods that best suited their selected goal and to identify potential obstacles.

Finally, to further reflect on gaps and opportunities, throughout the day, we zoomed out to discuss meta-issues for advancing prosocial intervention research. Again, we split up into groups to address this across several key themes: (a) mapping of research approaches for prosocial design, (b) reimagining collaborations with platforms, and (c) identifying collective projects to support independent research of prosocial design.

**Table S2**

*Participant list*

| **Category** | **Name** | **Affiliation** |
| --- | --- | --- |
| **Organizers** | David Grüning | PDN & University of Heidelberg |
|  | Julia Kamin | PDN & Civic Health Project |
|  | Emily Saltz | Jigsaw - Sr. UXR |
|  | Beth Goldberg | Jigsaw - Head of RSD |
|  | Rachel Xu | Jigsaw - Research Mgr |
|  | Thea Mann | Jigsaw - UX Design |
|  | Tin Acosta | Jigsaw - Product Manager |
|  | Zaria Howard | Jigsaw - Engineer |
| **Panelists** | Amy Bruckman | Georgia Institute of Technology |
|  | Dominic DiFranzo | Lehigh University |
|  | Max Allamong | Duke University |
|  | Kylan Rutherford | Civic Health Project & Columbia University |
| **Industry & Civil Society** | Alex Leavitt | Roblox & University of California, Berkeley |
|  | Becca Ricks | Mozilla |
|  | Jeanette Leagh | New York Times |
|  | Nan Noble | Reality Team |
|  | Sarah Ingle | New__Public |
| **Academia** | Anshuman Chhabra | University of California, Davis |
|  | Filippo Menczer | Indiana University |
|  | Jason Radford | Northeastern University |
|  | Jay Van Bavel | New York University |
|  | Joshua Tucker | New York University |
|  | Jane Im | University of Michigan |
|  | Kaylin Dodson | Brown University |
|  | Magdalena Wojcieszak | University of California, Davis & University of Amsterdam |
|  | Matthew Katsaros | Yale University |
|  | Natalie Bazarova | Cornell University |
|  | Smitha Milli | Cornell University |
|  | Talia Stroud | University of Texas at Austin |
|  | Tyler Musgrave | University of Michigan |
|  | Yixue Wang | Northeastern University |

**Table S3**

*Categorization of identified next actions to take for collaborative digital intervention research*

| **Category** | **Theme** | **Description** |
| --- | --- | --- |
| Knowledge and Resource Sharing | Best Practices Exchange | Create a repository for researchers to share best practices for conducting ecologically valid research of prosocial digital interventions. |
|  | Code Repository & Open Source Platform | Create a repository for sharing code and build an open-source platform for social media research tools. |
|  | Open Source Norms | Develop and promote open sourcing norms for sharing tools, methods, and datasets. |
|  | Researcher Guide for Platform Collaboration | Write a guide for new researchers on how to collaborate with platforms. |
| Events | Research Practices Event | Host a follow-up event in 2024 focused on sharing knowledge around research practices. |
|  | Research Tools Hackathon | Organize an event focused on building tools and solutions for research challenges. |
| Funding & Infrastructure to Support Collaborative Research | TESS-like Collaborative Research Platform | Fund and build infrastructure for conducting large-scale, ecologically valid research studies. |
|  | SDC-like Collaborative Research Competition | Fund and build infrastructure for a competition to conduct effective, jury-selected studies. |
|  | Funding for AI Misinformation Research | Fund a joint project to develop AI for debunking misinformation. |
| Other ideas | Data (DSA Request) Commons | Collectively request and advocate for platform data access (APIs, tools, etc.). |
|  | Term Standardization | Create a working group to define common terminology across fields. |
|  | Research Consortium | Advocate for legal support for conducting TOS-violating research. |
|  | Research Training | Develop courses to train researchers, focusing on understanding users as people. |
|  | Translate Research for Individuals | Make research findings accessible to individuals and promote media literacy. |
|  | Translate Research for Tech Professionals | Train tech professionals to build prosocial platforms and interventions. |
|  | Data Scrapability Regulation Proposal | Advocate for regulation to ensure all data can be scrapped, with users deciding data privacy settings. |
|  | Pre-registration archive | Create a prosocial pre-registration preprint archive. |
|  | Research challenge | How to study multi-platform interventions, also with off-platform data. |
